# Evaluating the Emotion Ontology through use in the self-reporting of emotional responses at an academic conference

**DOI:** 10.1186/2041-1480-5-38

**Published:** 2014-09-03

**Authors:** Janna Hastings, Andy Brass, Colin Caine, Caroline Jay, Robert Stevens

**Affiliations:** Cheminformatics and Metabolism, EMBL – European Bioinformatics Institute, Wellcome Trust Genome Campus, Hinxton, CB10 1SD UK; Swiss Center for Affective Sciences, University of Geneva, Geneva, Switzerland; School of Computer Science, University of Manchester, Oxford Road, Manchester, M13 9PL UK; Faculty of Life Sciences, University of Manchester, Oxford Road, Manchester, M13 9PL UK

## Abstract

**Background:**

We evaluate the application of the Emotion Ontology (EM) to the task of self-reporting of emotional experience in the context of audience response to academic presentations at the International Conference on Biomedical Ontology (ICBO). Ontology evaluation is regarded as a difficult task. Types of ontology evaluation range from gauging adherence to some philosophical principles, following some engineering method, to assessing fitness for purpose. The Emotion Ontology (EM) represents emotions and all related affective phenomena, and should enable self-reporting or articulation of emotional states and responses; how do we know if this is the case? Here we use the EM ‘in the wild’ in order to evaluate the EM’s ability to capture people’s self-reported emotional responses to a situation through use of the vocabulary provided by the EM.

**Results:**

To achieve this evaluation we developed a tool, EmOntoTag, in which audience members were able to capture their self-reported emotional responses to scientific presentations using the vocabulary offered by the EM. We furthermore asked participants using the tool to rate the appropriateness of an EM vocabulary term for capturing their self-assessed emotional response. Participants were also able to suggest improvements to the EM using a free-text feedback facility. Here, we present the data captured and analyse the EM’s fitness for purpose in reporting emotional responses to conference talks.

**Conclusions:**

Based on our analysis of this data set, our primary finding is that the audience are able to articulate their emotional response to a talk via the EM, and reporting via the EM ontology is able to draw distinctions between the audience’s response to a speaker and between the speakers (or talks) themselves. Thus we can conclude that the vocabulary provided at the leaves of the EM are fit for purpose in this setting. We additionally obtained interesting observations from the experiment as a whole, such as that the majority of emotions captured had positive valence, and the free-form feedback supplied new terms for the EM.

**Availability:**

EmOntoTag can be seen at http://www.bioontology.ch/emontotag; source code can be downloaded from http://emotion-ontology.googlecode.com/svn/trunk/apps/emontotag/and the ontology is available at http://purl.obolibrary.org/obo/MFOEM.owl.

**Electronic supplementary material:**

The online version of this article (doi:10.1186/2041-1480-5-38) contains supplementary material, which is available to authorized users.

## Background

Ontology evaluation is the assessment of how good an ontology is for one or multiple purposes [1]. Biomedical ontologies are being developed to address multiple requirements in biology and medicine including standardisation, data annotation and statistical analysis [2]. Ontology evaluation is recognised to be a difficult problem [1], with modes of evaluation ranging from conformance to some philosophical principle [3, 4], adherence to a specified method [5], conformance to a corpus of text or data [6], to ‘fitness for purpose’ for a given task [7].

Formal evaluations of biomedical ontologies are rare and this paper presents an evaluation of an ontology vocabulary’s ability to make the distinctions necessary in a field of interest. To this end, we report on an evaluation of the suitability of the Emotion Ontology (EM, [8, 9]) ‘in use’ for the self-reporting of emotional experiences at an academic conference. As the ontology has previously been described in [9] we do not here repeat that material. Rather, we focus on describing our experiment in which in order to assess the emotional vocabulary of the EM’s ‘fitness for purpose’ for the self reporting of emotional experience, we used the ontology’s vocabulary to capture an audience’s emotional responses to academic presentations at the International Conference on Biomedical Ontology (ICBO) [10] that was held in Graz, Austria in July 2012. We conducted this evaluation through the development of a tool, EmOntoTag, by means of which audience members were able to capture their emotional responses to the scientific presentations using the vocabulary offered by the EM.

An ontology makes distinctions between entities in a field of interest. In our case, the EM makes distinctions between types of emotions, such as being bored or interested. In an academic conference we can assume that neither talks nor the audience are homogeneous in emotional response provoked or elicited. In the biomedical ontology community in particular, there are well known contentious approaches to ontology engineering [3, 11]. Thus we can expect that different talks will provoke different emotional responses and that audience members wil have a range of differing emotional responses to talks at ICBO 2012. From this, our null hypothesis (H0) is:

The EM will not enable audience members to articulate their emotional response to a talk appropriately such that we can cluster the audience by their response to a talk. We will find that people and talks are not able to be distinguished by the descriptions of emotional responses.

If the null hypothesis is rejected, we may expect conference participants to be able to use the EM to articulate an emotional response to a talk and that talks and the audience can be partitioned by emotional response.

To test this hypothesis, we allowed audience members to give their emotional responses to talks using the vocabulary drawn from the ontology, and also asked them to rate how appropriate an EM vocabulary term was for articulating an emotional response.

Examples of the phrases that were used to capture emotions during the conference include ‘I feel *interested*’, ‘I feel *bored*’ and ‘I think that *this is being caused supernaturally*’. The rating given by users as to how easy it was to use the EM to capture their emotions ranged from 1 (it was difficult to capture the emotion being experienced) to 5 (easy to capture the emotion being experienced). We also asked participants to suggest improvements to the ontology’s content using a free-text feedback facility; the aim here was to capture emotions that participants felt they could not articulate using the EM.

We were also able to collect the following information:

-  Which vocabulary terms were used and with what frequency;
-  The numbers of terms used per talk;
-  The time at which a term was used and by which (anonymous) audience member;
-  The number of people participating in the study;
-  The strength of emotional response to talks.

While the evaluation of ontologies in use in applications have been conducted before (as discussed in [1, 12]; for a recent example evaluating the Gene Ontology in use see [13]), we believe that the approach we have followed of combining the use of an ontology in an application with the simultaneous rating of the ease of use of the ontology’s vocabulary for that application is a novel technique that could have applicability outside the scope of the present investigation.

### The Emotion Ontology

Capture of emotional experience is a component of a variety of different research and application scenarios. For example, self-reported emotional experiences are often captured to monitor mood fluctuations between clinical visits in the clinical treatment of depression and bipolar disorder [14, 15]. Self-reported emotional experiences may also be useful in the assessment of response to software tools, new products, or audience response to academic presentations. Various tools have been developed that allow capture of emotional experience in the context of specific application needs (e.g. [16, 17]). However, there has been no agreement on shared identifiers for the underlying structure of the emotional domain such that annotations could be compared between different tools and across different projects that employ different levels of specificity.

Ontologies provide a flexible hierarchically organised structure for defining entities and vocabulary within a domain [18], and have been highly successful in enabling standardisation in biomedical contexts [2]. Reflected in generic ontology languages such as the Web Ontology Language (OWL, [19]), ontologies are computable and supported by many open source libraries across multiple languages, thus are suitable for implementation into a wide range of different tools. Reuse of a shared ontology across multiple tools enables subsequent aggregation and comparison between annotations arising from heterogeneous projects [20], as has been amply illustrated by successful applications of the Gene Ontology project [21, 22].

The Emotion Ontology (EM) is an ontology being developed for the domain of the emotions and all related affective phenomena [8, 9]. The ontology aims to address diverse requirements arising from the full range of disciplines involved in research into affective phenomena, including psychology, psychiatry, neuroscience, biomedicine and the life sciences. Such applications include standardised data annotation for aggregation across databases, meta-analyses of primary research results, mapping across disciplines for translation of primary research into candidate therapeuticals, semantic searching and querying of literature and databases such as implemented by the Neuroscience Information Framework [23], and automated text analysis for addressing the semi-automatic curation of the vast quantities of scientific literature [24]. We have previously used the ontology in the automatic detection of emotions in the text of suicide notes, with potential application to the analysis of the diary writings of suicide-risk patients to assist in suicide prevention measures [25].

The EM currently consists of distinct branches for emotions and related phenomena. As is documented in the metadata of the ontology, the vocabulary included in the EM ontology has largely been drawn from [26] and the vocabularies used in the GRID cross-cultural project [27]. The upper-level structure of the ontology, as reported in [9], distinguishes emotions *proper* as complex processes, for example anger or fear, from other physiological and mental processes that may form a part of an emotion process, including cognitive appraisal processes and subjective feelings. From this complex structure, we identified three branches of the ontology that we believed to be of relevance for the self-reporting of emotional experiences: appraisals (cognitive judgements that may trigger emotions), subjective feelings (inner awareness of affective feelings), and emotions proper.

For example, *anger* is an emotion defined in the EM as “Anger is a negative emotion, characterised by feelings of unpleasantness and high arousal, in the form of antagonistic feelings and action tendencies,” and *fear* is defined as “An activated, aversive emotion that motivates attempts to cope with events that provide threats to the survival or well-being of organisms. Characterised by feelings of threat and impending doom, and by an urge to get out of the situation”. *Feeling restless* is a subjective feeling defined in the EM as “The subjective emotional feeling of restlessness, a state of not being calm, of an agitation to do something”.

The other branches of the ontology, including behavioural responses to emotions such as facial expressions and physiological responses to emotions such as an increased heart rate, were excluded from this experiment by virtue of these not being appropriate to the use case of self-reporting of emotional experience.

## Methods

We used self-reporting of emotional response to the talks at ICBO 2012 and our approach had the following components:

1. Design and implementation of a Web application (EmOntoTag) that enables users to anonymously login, ‘tag’ their emotional response to an ICBO 2012 presentation using terms from the EM, and record how appropriate they felt an EM term was at articulating an emotional response.
2. Obtaining permission from presenters for their presentation at ICBO to be included in this study.
3. Running the experiment during the ICBO conference on July 23–25 2012, and analysis of the data obtained.

### Design and development of EmOntoTag

We conceived a tool that would be light-weight and able to run in any Web browser to enable the broadest range of conference attenders to participate in the experiment. The primary requirement that we identified for this tool was that it would allow the user to self-report their current emotional experience with a minimum of overhead, such as technical terminology or excessive clicking. Furthermore, in order to address the hypothesis we forced the user to capture how well the EM’s vocabulary was able to capture the respondant’s emotional response as a rating attached to every record they made of their emotional experience with this tool.

A Web tool was implemented in the Python language on top of a MySQL database. This was subsequently wrapped with the Jython Java–Python bridge to enable deployment in a Tomcat Web application server. All source code for the implementation of EmOntoTag is available from the repository hosted at [8]. EmOntoTag can be accessed via http://www.bioontology.ch/emontotag with login ‘guest’, for which responses will not be recorded.

In order to anonymise the users of the tool, while still controlling access in order to ensure that we could distinguish different users’ responses, we prepared anonymous random access codes printed on sheets of paper which were handed out to conference participants by the conference organisers. Only the anonymous random codes were stored against the tags in the underlying database. An example of the sheet handed to conference participants is available as Additional file 1.

After obtaining an access code and logging into the tool, users were presented with the list of available presentations linked to information about the conference schedule. The tool had a register of the conference schedule and was able to direct users to the currently ongoing presentation (at least according to the conference schedule). Presentations were indexed by author names and by presentation title. Only those presentations for which the presenter agreed in advance to participate in the experiment were enabled in the tool; for those presentations for which the presenter did not agree, the tool showed a message that the presentation was not available for tagging.

For each presentation, the users were offered a response capture interface that allowed them to articulate their emotional response using the vocabulary from the underlying EM ontology. As described in [9], EM distinguishes emotions “proper”, i.e. full complex emotional experiences associated with an object, from subjective feelings, which are simpler feelings and which don’t necessarily have an object, and appraisals, which are the cognitive (thought) component of emotions which are viewed in some theories to be the triggers of emotions [28]. As a design choice to enable ‘natural’ emotional expression, options were provided to the user in the context of sentence completion, where the allowed sentences began with ‘I feel’ and ‘I think’. ‘I feel’ was used as the precursor to the selection options from the ‘emotion’ and ‘subjective feeling’ branches of the ontology, while ‘I think’ was used as the precursor to the appraisal (cognitive) branch of the ontology. To accommodate the fact that the labels for emotion terminology in the ontology were in the noun form, e.g. ‘fear’, additional synonyms were added to the ontology that would fit better in the context of a sentence, e.g. ‘afraid’.

Examples of the sentences that were available for expression of emotions include:

-  ‘I feel *interested*’,
-  ‘I feel *despairing*’,
-  ‘I feel *calm*’ and
-  ‘I think that *this is familiar*’.

**Figure 1:**
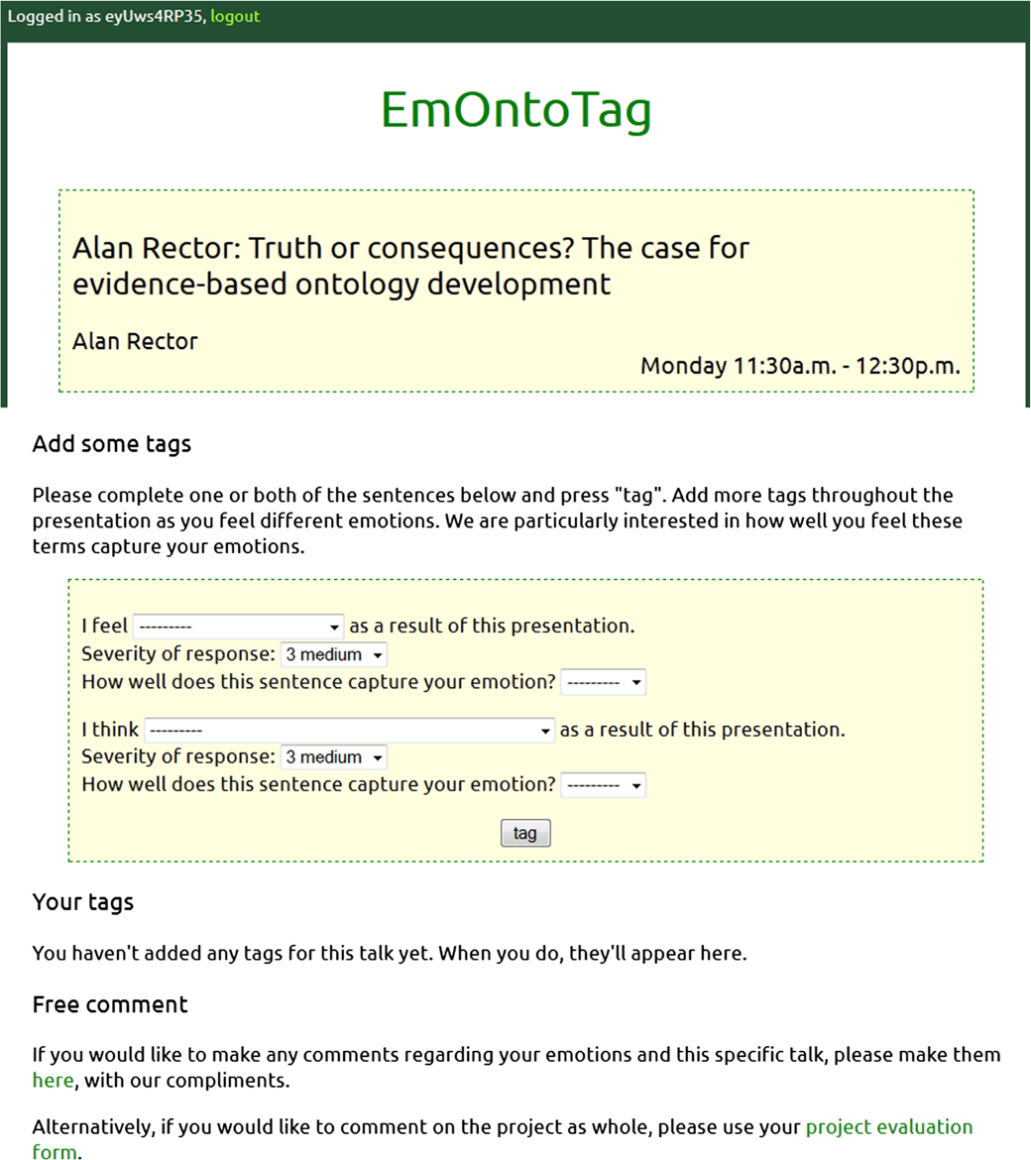
**Screenshot of EmOntoTag facility for capturing emotional experience.**

The full list of options that were provided in the drop-down selections in the tool interface are provided in Table 1.Options for sentence completion were presented in a random sequence, not sorted alphabetically, in order to avoid bias towards certain terms. This almost certainly reduced usability of EmOntoTag, but our desire to avoid too much bias over-rode this usability issue. Figure 1 shows a screenshot of the EmOntoTag user interface for selection of an ‘EM sentence’ during the experiment.

**Table 1:** **Emotion Ontology vocabulary used in the experiment**

Emotion Ontology vocabulary		
**Emotion**	**Subjective feeling**	**Appraisal**
**Prefix: ‘I feel’**	**Prefix: ‘I feel’**	**Prefix: ‘I think’**
Surprised	Out of control	This is not expected
Happy	Good	I am being treated justly
Mastery	At ease	This is not predictable
pleasure		
Passionately	In control	This is not being deliberately
loving		caused
Sensory	Exhausted	This is dangerous
pleasure		
Disgusted	Energetic	A response is needed urgently
Grieving	Tired	This is being deliberately caused
Furious	Restless	This is not dangerous
Amused	Weak	This is expected
Despairing	Bad	I am not at the centre of attention
Jealous	Strong	There are consequences and they
		are unavoidable
Embarrassed	Nervous	I am being treated unjustly
Serene	Calm	This is not familiar
Terrified	Alert	This is not important for my goals
Irritated		This is familiar
Proud		This is being caused by chance
Interested		This is pleasant
Sad		I have irrevocably lost something
		important
Elated		This is against my ideals
Loving		This is predictable
Stressed		This is being caused by me
Sexual pleasure		This has undesirable consequences
Aesthetic pleasure		This is in line with my ideals
Compassionate		This is unpleasant
Euphoric		There are consequences but they
		are avoidable
Social pleasure		This is being caused supernaturally
Anxious		This is important for my goals
Enraged		This has desirable consequences
Bored		This is not sudden
Contemptuous		This is being caused by someone
		else
Pleasure		A response is needed but not
		urgently
Ashamed		This is sudden
Panicked		I am at the center of attention
Hateful		
Angry		
Contented		
Disappointed		
Guilty		
Joyful		
Afraid		
Compassionately loving		

The table gives a listing of the vocabulary drawn from the Emotion Ontology that was provided to users of the EmOntoTag tool during the conference.

Having specified a sentence describing the emotional experience at that moment, the user was required to rate how well the vocabulary provided by the EM captured their emotional response. They were also able to offer a strength of response for the EM sentence used. All the user’s previously captured sentences for that talk were displayed in a table lower on the screen, indexed by the time of capture, and it was possible to delete previous sentences, to allow for the correction of errors. Deleted sentences were not used in the subsequent analysis.

It was also possible to use a separate free text input field to record requests for content for the EM or problems with the EmOntoTag user interface. We used this as the means to gather information about possible extensions to the EM.

### Obtaining permission to run the experiment

We obtained permission from the presenters before including their presentations in the experiment. Presenters were contacted individually by email in order to request permission to include their presentation in the experiment, and their permission was sent by reply email. Only the scientific presentations were included in the experiment, together with the two invited keynote talks. The response was overwhelmingly positive; from the 26 papers and 2 keynote talks: all but one paper presenter gave their permission for inclusion in the experiment. Audience participation was anonymous, with no realistic way of tracing alphanumeric login codes to any individual. Actual participation in recording emotional response was voluntary. All data were stored securely. We will not report here on which responses were made for which particular talks, with the exception of a selected talk for which we obtained specific permission.

### Experiment execution and data analysis

We enabled the EmOntoTag software on the weekend before the conference was due to start, and announced and explained the experiment during the opening session of the conference on the morning of July 23^rd^ 2012. While the tool is still available online to enable interested parties to examine the interface, the cutoff date for responsses which we included in the experiment was set at 27^th^ July 2012, i.e. 2 days after the conference closed. This excluded two extremely late tags, but allowed for ‘slow’ responses.

The dataset was analysed using the R statistical analysis package and Matlab. We aggregated all the data into a data table indexed by anonymous user ID, ontology term ID, time of response, talk ID, strength of response, and appropriateness of the EM’s content. Furthermore, the ontology terms were grouped by their valence into three categories – positive, negative and neutral.

To test the hypothesis that emotional response can be partitioned into those for talks and those for the audience, we took these raw data and created two tables:

1. One capturing users by the EM terms they had used;
2. One capturing talks by how they were described.

These tables were then normalised (so that the sum of entries was one) to allow for the variation in number of EM terms used to describe talks and in the numbers of talks to which different users had responded.

These two tables were then analysed using a principal components strategy to determine which linear combinations of terms described the greatest variation in responses of people and EM terms. This gave us a set of eigenvalues and eigenvectors which could be used to describe the data.

## Results

Raw data are not provided in order to protect confidentiality.

### Number of respondants and EM terms used

**Figure 2:**
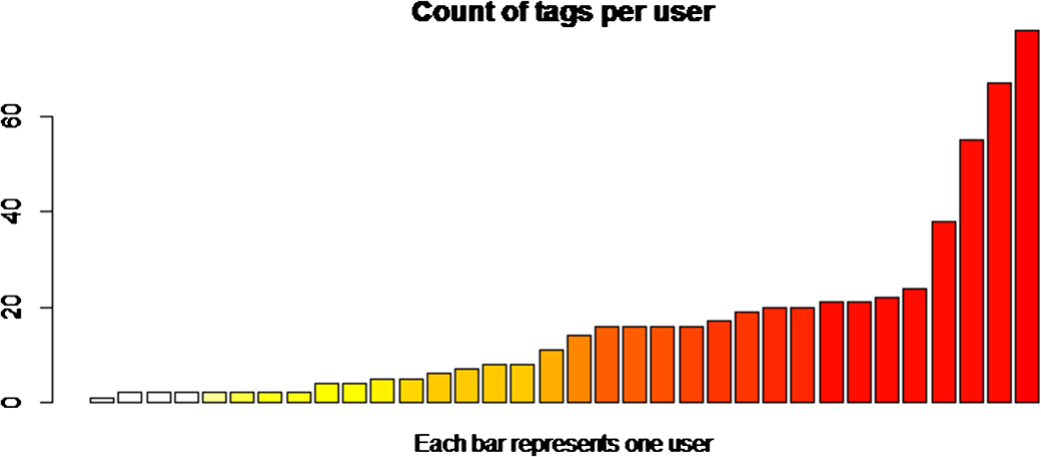
**The counts of responses per participating user.**

The total number of EM terms captured in the experiment was 553, spread across the 27 presentations that agreed to participate in the study (25 paper presentations and 2 keynotes). Of these, all 27 had at least 4 EM terms captured in the experiment, and the largest number of terms captured against one talk was 67. There were 35 distinct users from the 80 registered conference attenders (44%) who captured EM terms during the experiment. Of these, the range of numbers of responses was large, with the most active user providing 78 terms and the least active user providing one term. The number of responses per user is shown in Figure 2.

The full set of counts for users and usages per term type is given in Table 2.

**Table 2:** **Number of users and tags**

Term id	Term label	Number of tags	Number of users	Valence	Type
33	Interested	86	28	Positive	Emotion
42	Happy	30	18	Positive	Emotion
166	Bored	26	13	Negative	Emotion
68	This is expected	25	12	Neutral	Thought
44	Amused	22	12	Positive	Emotion
64	This is familiar	20	13	Neutral	Thought
73	This is important for my goals	19	12	Positive	Thought
12	Annoyed	19	8	Negative	Emotion
70	This is pleasant	17	13	Positive	Thought
95	This is in line with my ideals	17	11	Positive	Thought
86	This has desirable consequences	16	13	Positive	Thought
11	Irritated	16	7	Negative	Emotion
80	Tired	14	8	Negative	Feeling
111	Restless	13	8	Negative	Feeling
37	Mastery pleasure	11	3	Positive	Emotion
47	Contented	10	6	Positive	Emotion
114	Calm	9	6	Neutral	Feeling
41	Proud	9	4	Positive	Emotion
66	This is predictable	8	7	Neutral	Thought
51	Disappointed	8	6	Negative	Emotion
84	This is not being deliberately caused	8	4	Neutral	Thought
74	This is not important for my goals	7	7	Neutral	Thought
65	This is not familiar	7	6	Neutral	Thought
32	Surprised	7	5	Neutral	Emotion
43	Serene	7	4	Positive	Emotion
124	Nervous	6	6	Negative	Feeling
79	Good	6	6	Positive	Feeling
71	This is unpleasant	6	6	Negative	Thought
35	Pleasure	6	5	Positive	Emotion
101	I am being treated justly	6	4	Positive	Thought
34	Joyful	6	4	Positive	Emotion
121	Alert	6	3	Positive	Feeling
30	Despairing	5	5	Negative	Emotion
109	Energetic	5	5	Positive	Feeling
67	This is not predictable	5	4	Neutral	Thought
92	There are consequences but they are avoidable	4	4	Neutral	Thought
96	This is against my ideals	4	4	Negative	Thought
107	At ease	4	4	Positive	Feeling
52	Compassionate	4	4	Positive	Emotion
56	Sad	4	3	Negative	Emotion
46	Euphoric	4	3	Positive	Emotion
83	This is being deliberately caused	4	2	Neutral	Thought
39	Aesthetic pleasure	3	3	Positive	Emotion
87	This has undesirable consequences	3	2	Negative	Thought
69	This is not expected	3	2	Neutral	Thought
36	Sensory pleasure	3	1	Positive	Emotion
40	Sexual pleasure	2	2	Positive	Emotion
28	Anxious	2	2	Negative	Emotion
54	Embarrassed	2	2	Negative	Emotion
62	This is sudden	2	2	Neutral	Thought
19	Disgusted	2	2	Negative	Emotion
76	This is being caused by me	1	1	Neutral	Thought
81	This is being caused supernaturally	1	1	Neutral	Thought
93	There are consequences and they are unavoidable	1	1	Neutral	Thought
102	I am being treated unjustly	1	1	Negative	Thought
110	In control	1	1	Positive	Feeling
116	Out of control	1	1	Negative	Feeling
13	Furious	1	1	Negative	Emotion
50	Passionately loving	1	1	Positive	Emotion
55	Ashamed	1	1	Negative	Emotion
90	A response is needed but not urgently	1	1	Neutral	Thought
112	Exhausted	1	1	Negative	Feeling
26	Afraid	1	1	Negative	Emotion
78	This is being caused by someone else	1	1	Neutral	Thought
89	A response is needed urgently	1	1	Neutral	Thought
106	I have irrevocably lost something important	1	1	Negative	Thought
119	Weak	1	1	Negative	Feeling
9	Angry	1	1	Negative	Emotion

Results table with count of usages per term, with distinct users, valence and type. The table gives the numbers of users and tags for each of the tag types that was used by participants in the experiment.

### Ease of articulating emotional response

The rating of how well the EM vocabulary allowed the user to articulate their emotional response had a mean of 3.42, with standard deviation 1.12. This significantly differed from the median of 3; i.e. the users reported, on average, that the vocabulary did allow them to capture their emotions well (*t* = 8.7324,*df* = 552,*p* < 2.2^-16^).

Our result can further be decomposed by grouping the responses per ontology term type. There were three different types of ontology term used in this experiment: appraisal (thoughts), subjective feeling and emotion. Of these, in fact, the emotions have the highest mean and the highest significance, while the thoughts category, with a mean of 3.14, was not significantly different from the median of 3 (Thoughts: mean = 3.14, *t* = 1.6859,*df* = 187,*p* = 0.09348; Feelings: mean = 3.37, *t* = 2.7126,*df* = 66,*p* = 0.008505; Emotions: mean = 3.60, *t* =9.7564,*df* =297,*p* < 2.2^-16^).

### Strength of response

The mean of the strength of response is 3.07, standard deviation 0.92. In contrast to the appropriateness of an EM term, the mean strength of response does not differ significantly from the median of 3 (two-tailed *t* = 1.8007,*df* =552,*p* = 0.0723).

### Valence of responses

**Figure 3:**
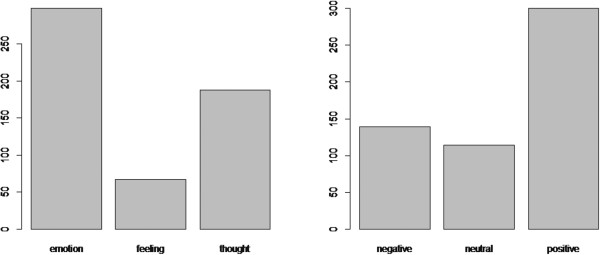
**Counts of response by valence and by ontology term.**

The ontology terms were separated into three categories according to their valences: positive, negative, and neutral. Neutral was used for emotions such as *surprise* which are known to have either positive or negative valence, and for many of the appraisal categories in which the same applied.Based on this division, we found that the majority of responses were positive (300 positive, 139 negative, 114 neutral). Figure 3 shows the counts of terms used per ontology type and per valence.

Positive responses were also found to have been rated as stronger, i.e., having a greater strength of response rating. Indeed, while no significant effect was detected for the overall rating of strength of response and also not for the neutral or negative response groups, the positive responses were significantly stronger than the mean (mean of strength of response (neutral): 2.991228; mean (positive): 3.186667 (*t* = 3.6273,*df* = 299,*p* = 0.0003366); mean (negative): 2.884892).

The positive responses also obtained a slightly better appropriateness score than the negative or neutral responses (mean of appropriateness (neutral): 3.210526; mean (positive): 3.386667; mean (negative): 2.654676). The negative mean is significantly different to the positive mean: *t* = 2.3365,*df* = 253.678,*p* = 0.02024, while the positive mean is not significantly different to that for neutral responses (*p* = 0.1734). The negative mean is also significantly different to that for neutral responses: *t* = 2.9837,*df* = 235.493,*p* = 0.003147.

### Usage of ontology terms

**Figure 4:**
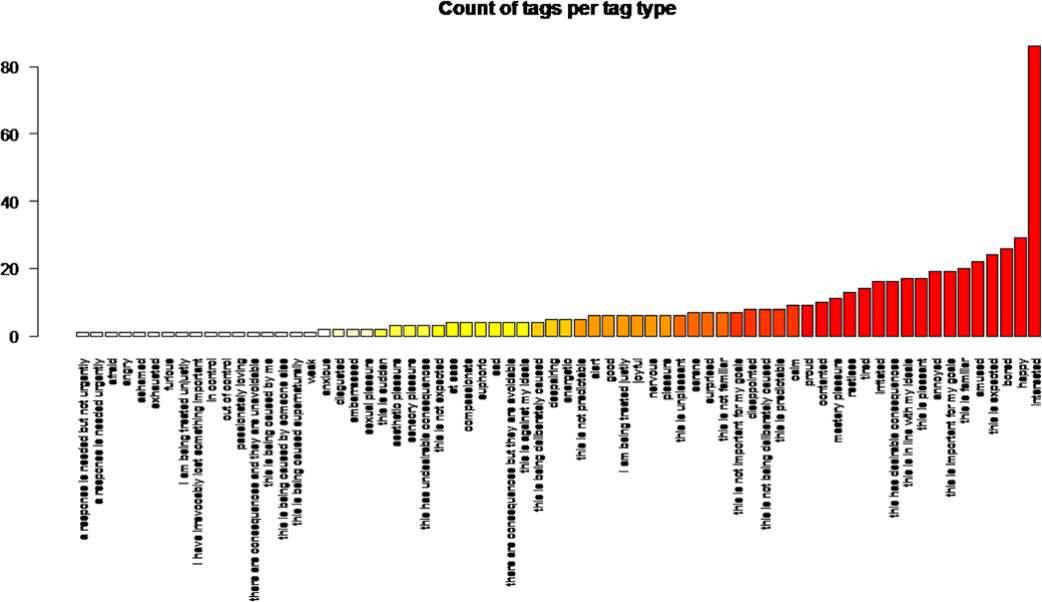
**Counts of tags for each ontology term used (non-zero occurrence).**

Of the total of 89 ontology terms that were included in the study, 67 were actually used. The most commonly used term was ‘interested’, with 86 occurrences. The distribution of counts per ontology term is shown in Figure 4.

The number of EM terms used per talk varies, with the highest being 67 and the lowest 4. There was a spike in responses during the first talk (58) and thereafter fewer in general for subsequent talks, with spikes at the two keynotes and a resurgence in the last day. The two keynotes gave sharp increases in the number of EM terms captured relative to the remainder of the talks (67 and 30), which also makes sense given that the time the speakers were talking was much longer. The second day had the lowest number of responses, with a bit of a revival on the last day for the last three talks.

The mean number of EM terms used per talk was 20.48, median 17 and standard deviation 15.48.

### Can talks and audience members be distinguished by EM terms?

An analysis of the eigenvalues (scree plots) showed that the data could not be readily embedded in a low dimensional space. For both data sets, the first 5 eigenvectors combined only captured 75% of the variation in the data. This is unsurprising in a domain with such inherent complexity as an academic audience’s emotional response to a series of research presentations; in contrast, an embedding into a low-dimensional space would have been surprising.

However, looking at the EM terms that dominated the first five eigenvectors in these two sets shows that there were differences between talks and the audience. The ordering of key emotional terms needed to account for the varience in the talks and the audience are shown in Table 3 in descending order of strength (including the counts of usage).

**Table 3:** **Terms that most describe users compared with terms that most describe talks**

	Talks partition					Audience partition			
Term	Type	Valence	Id	Count	Term	Type	Valence	Id	Count
Interested	Emotion	Positive	33	86	This is pleasant	Thought	Positive	70	17
Restless	Feeling	Negative	111	13	Happy	Emotion	Positive	42	29
Bored	Emotion	Negative	166	26	This is familiar	Thought	Neutral	64	20
Annoyed	Emotion	Negative	12	19	Interested	Emotion	Positive	33	86
Happy	Emotion	Positive	42	29	This is expected	Thought	Neutral	68	24
Amused	Emotion	Positive	44	22					

Eigenvectors of terms that most describe the audience compared with terms that most describe talks; ordered in descending strength. The table gives the terms that best describe users and the terms that best describe talks.

### Free text comments

The free text comments yielded several suggestions of terms that were missing from the ontology at the time of the experiment. These were:

1. Curious (requested twice)
2. Concerned
3. Dubious
4. Worried
5. Confused (requested four times)
6. Distracted or unfocused
7. Indifferent, emotionally neutral or feeling nothing (requested three times)
8. Expectant or anticipative
9. Hopeful
10. Inspired (requested twice)
11. Intrigued
12. Schadenfreude.

As a concrete outcome of this experiment, almost all of these missing emotional terms have been added to the ontology. The exceptions are ‘distracted or unfocused’, which was deemed not to be an emotion term *per se* but rather having relevance to attention, which will be covered in the context of the broader Mental Functioning ontology project [24], and ‘emotionally neutral or feeling nothing’, which again was not considered to be an emotion but rather the absence of an emotion. The latter case, emotionally neutral, should however be added as an option provided by the user interface in subsequent versions of the EmOntoTag tool. Additionally, ‘amused’ was requested, despite this term actually being available in the list of options.

Some suggestions were received via the free text comment facility for alternative phrasing for certain of the listed emotions and feelings, for example *why can’t I just say ‘pleased’?* and *‘feeling of mastery’ better than ‘mastery pleasure’*. These suggestions have been incorporated into the ontology by updating the ‘tag display’ synonyms for the relevant terms.

A small number of comments related to the usability of the EmOntoTag tool employed in the experiment, specifically: *Sometimes I get the red warning, ‘This field is required’, sometimes not, for what is apparently the same behaviour*, and *Why did you not list the emotions alphabetically?*. The choice of unsorted presentation of selection options was done to avoid bias, though it does have an obvious usability penalty. By verbal communication, another comment that we received as feedback on the usability of the tool was that it was not optimised for smart phone and other smaller screens. These enhancements will be incorporated into subsequent versions of the tool.

Finally, several comments requested that the appraisal or thought list was not specific enough because it did not allow the specification of the actual cause of the emotion in question. The appraisal list included *generic* appraisal components such as *I think that there will be consequences* or *I think that this is being supernaturally caused*. Participants, on the other hand, used the free text to request the ability to express the *specific* cause of their emotion, for example *I feel bored because I have heard this all before*, *I was surprised that what I thought was an important aspect of the topic was missing from the talk*, and *I was afraid he would run out of time*. Those causes that are reflected in the vocabulary of the EM are derived from those that have been found to be fairly generic (i.e. applicable across multiple scenarios) in the cross-cultural GRID project that investigated the meaning of emotion terms [27]. Clearly, the EM cannot include a vocabulary for all the possible scenarios and objects that can cause an emotion. The accurate description of the objects of the emotion and the way that these objects are intricately linked to the type of the emotion will be the subject of future Emotion Ontology development.

### Exploring the emotional response to one talk

**Figure 5:**
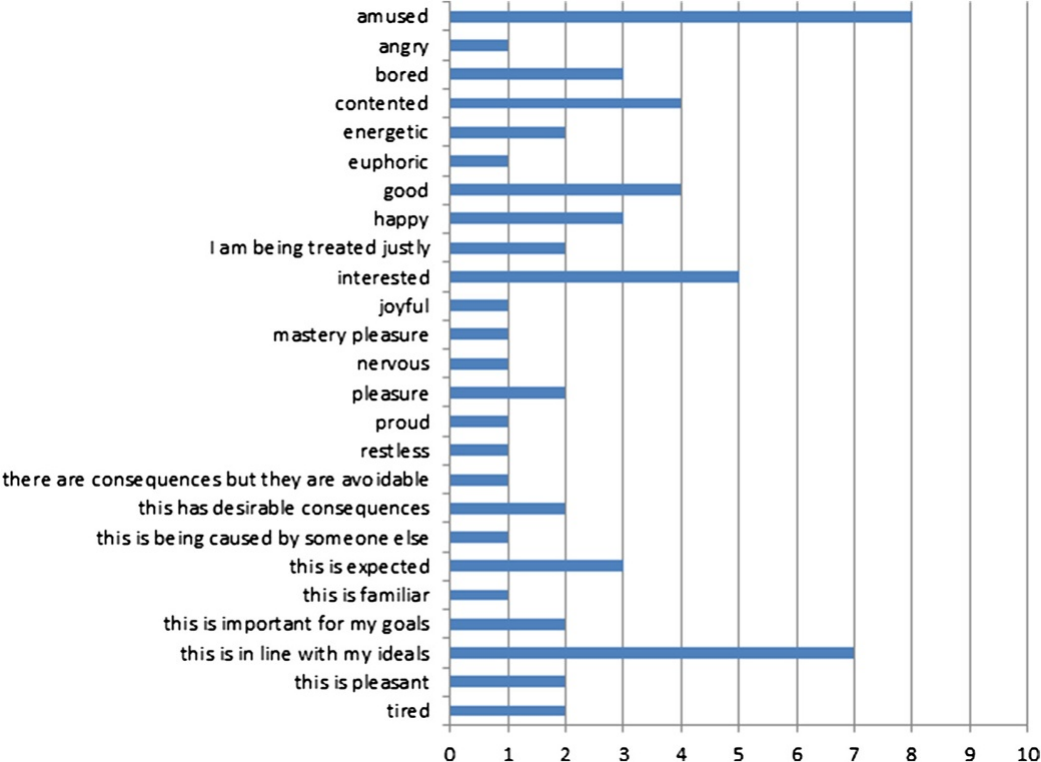
**The EM terms used to articulate the emotional response to one of the ICBO 2012 talks; y-axis are the terms used and x-axis is the number of times each term was used.**

**Figure 6:**
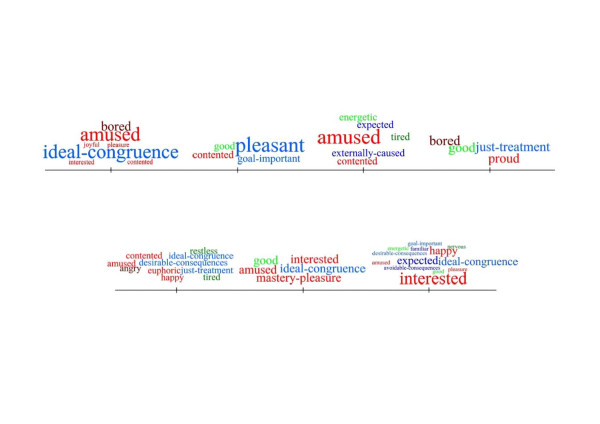
**A time-line of emotional responses to the sample talk; the EM terms are put into bins and displayed as tags, the size of which is proportional to the number of times the tag was used.** The general area of the EM is indicated by colour: appraisals in blue, emotions in red and feelings in green. Valence is indicated by shading – darker for negative and lighter for positive. Exact times and durations are obscured to avoid the talk being identified.

We were given permission by one of the presenters to reveal the results of the audience’s emotional response to his/her talk. A bar chart summarising the responses to the talk can be seen in Figure 5. Fifteen participants responded; the highest number of responses was 19 (participant 125) and the lowest was 1 response (participant 79, who displayed ‘mastery pleasure’–the feeling of ‘mastery’ of the subject). Figure 6 shows the spread of emotional responses during the talk and just after the talk.

## Discussion

Whilst we may have the emotional terms used by participants, we do not know the motivation for the articulation of that emotion. So, the discussion that follows is somewhat speculative. Also, tying reporting events to events in the talks themselves risks identification of the talks, so this discussion is limited to generalities.

The main part of our null hypothesis that ‘the EM will not enable audience members to articulate their emotional response …’ can be rejected. This is given support by the scores for the rating of how well the given vocabulary sentences captured the emotion the audience member wanted to express; these rating scores were significantly higher than the median value. This alone indicates that the EM is sufficient to allow emotions to be articulated.

The following terms from the EM vocabulary ‘interested’, ‘happy’, ‘amused’, ‘this is familiar’, ‘this is expected’, ‘bored’, (all with a count greater than or equal to 20) are terms most responders have used for most talks. These are emotional responses one would expect to dominate in an academic conference, with an audience interested and sometimes bored, with much that is familiar or expected, with a good deal of happiness and amusement thrown into the mix.

The principle components strategy was used in the second part of our hypothesis, that the responses using the EM would be sufficient to cluster emotional responses about talks and the audience. The PCA determined which linear combinations of tags described the greatest variation in responses about the audience and talks, and gave us a set of eigenvalues and eigenvectors which could be used to describe the data. Table 3 (above) shows six EM terms that partition to talks and five EM terms that partition to the audience. The key emotional terms (identifiers shown in parentheses) needed to describe the talks were: ‘interested’ (33), ‘restless’ (111), ‘bored’ (166), ‘annoyed’ (12), ‘happy’ (42) and ‘amused’ (44).

Whereas those that best described the audience were: ‘this is pleasant’ (70), ‘happy’ (42), ‘this is familiar’ (64), ‘interested’ (33) and ‘this is expected’ (68).

As the order matters, terms ‘this is pleasant’ (70) and ‘happy’ (42) are the EM terms that strongly describe the audience, while the terms ‘interested’ (33) and ‘restless’ (111) were important for distinguishing the talks.

The six talk terms are five *emotions* and one *feeling*; with three positive valence emotions (‘interested’, ‘amused’ and ‘happy’) and two negative emotions (‘bored’ and ‘annoyed’) and one negative valence feeling (‘restless’).

The five EM terms for the audience (two emotions and three thoughts) were all either positive or neutral; two positive emotions (‘interested’ and ‘happy’) and one positive feeling (‘this is pleasant’), with two of the *thoughts* being neutral (‘this is familiar’ and ‘this is expected’).

Two of the EM emotions (‘interested’ and ‘happy’) partition to both talks and audience (with ‘interested’ being strongly in the talks partition) suggesting that overall the talks and audience provoke or cause happiness and interest. The audience’s emotional responses are either positive or neutral and the EM’s *feelings* are associated with the people articulating the emotions. Much of the ICBO content may well be either ‘expected’ or ‘familiar’ in some form to the audience; this is ‘to be expected’ at most conferences – ‘this is the kind of thing that would be expected from X’. The general positive response of the audience wil be discussed further below.

The ‘talks’ partition is, perhaps, more interesting; here some negative terms appear as well as ‘interested’ and ‘happy’ – we have ‘annoyed’, ‘bored’ and ‘restless’. That any negative terms appearing are associated with the talks, rather than the audience, is reassuring; it is the talks that cause annoyance, boredom and restlessness – while it is the audience that feel that the talk is familiar, is expected and is pleasant.

That terms partition sensibly between talks and audience, with each having high counts, together with the partition being readily explicable leads us to believe that the EM has the ability to enable the articulation of an emotional response (at least in this situation).

Our PCA analysis shows that the EM is sufficient to allow discrimination between audience members’ emotional responses to a conference talk. We can see emotional tags that are associated more strongly with the audience and emotional tags that are associated more with the talk itself. Overall, as the EM term’s partitioning makes sense in the context of an academic conference, it indicates that the EM is competent to support conference attenders to articulate their emotional response to a talk and thus further supports our hypothesis.

The commonest tags used were ‘interested’, used 86 times by all users at some point in the conference, and ‘happy’, used 29 times by 17 participants in 18 talks, presumably reflecting a general level of contentment with the conference’s material (‘contented’ was used 10 times by 6 participants in 5 talks; other tags of this kind can be seen in Table 2). ‘Interested’ is the dominant emotional response from the audience, which may well be expected in a conference about ontologies, which an audience of ontologists has chosen to attend.

‘Bored’ was used 26 times by 13 users in 14 talks; a related set of tags, ‘angry’, was used once; ‘annoyed’ was used 19 times by 8 participants in 13 talks; ‘furious’ was used once; ‘irritated’ was used 16 times by 7 participants in 10 talks. The tags are related in the context of the community itself: irritation, fury, annoyance, and anger may be felt by those with entrenched opinion in opposition to those of the speaker – or about bad science, though the two may not be unrelated in the minds of the participants. Members of the biomedical community will be familiar with the divisions that exist within the community on fundamentals of ontologies [3, 11] and these divisions might have been reflected in the participants’ responses. In addition, a conference in which all talks are of an equally high quality and are equally highly appreciated will be rare or non-existent.

‘This is expected’ was used 24 times by 11 participants in 13 talks. The straight-forward interpretation is that the participants in question were hearing what they expected from the speaker in question, either positively or negatively. On the positive interpretation, this tag could be grouped with the feeling of pride (if this is expressed as, for instance, a result of one’s work or oneself being mentioned in a positive light). Amusement was a response articulated by 12 participants, 22 times in 11 talks. The most straight-forward interpretation of this is that talks contained an element of humour and the audience responded to this humour. Participants may have articulated amusement as ‘schadenfreude’ (an emotion that was requested as an addition to the EM). However, taking the most straight-forward interpretation of this tag, we can observe that ICBO had a reasonable amount of humour in its talks.

Some of the tags not used were ‘compassionately loving’, ‘contemptuous’, ‘guilty’, ‘terrified’, ‘I am at the centre of attention’. It is posssible to conceive of ways in which these unused tags could have been used – a person singled out in a talk may feel to be the ‘centre of attention’, or being ‘guilty’ of an ontological crime highlighted by a speaker. Others, such as being ‘contemptuous’ are perhaps not required in this context when being ‘angry’ is available, despite the obvious differences. The tag ‘sexual pleasure’ was used once each by two participants. If a true reflection of response to either speaker or topic it may be disturbing, but it may also have been used in jest—despite this, the EM still enabled the emotion to be articulated.

There is a strong tendency towards EM terms with a positive valence being used. There are at least four posible factors involved:

1. The anonymity of reporting should allow negative as well as positive emotional responses to be reported;
2. Factions within the biomedical ontology community and variability in the quality of the presented work should mean there are negative emotional responses to talks;
3. Basic ideas of reduction in cognitive dissonance [29] may incline reported emotions to be positive; that is, audience members need to justify to themselves their presence at the conference – an individual giving a broadly negative emotional response would suggest he or she had attended the wrong conference. Similarly, acquiescence bias [30] leads to individuals tending to respond ‘yes’ or positively to questions or situations.
4. The ICBO audience is self-selecting and will be pre-disposed to liking talks about biomedical ontologies; so, in spite of factionalism in the community, most people will be emotionally positive most of the time.

Points three and four seem to have out-weighed points one and two. In addition, point one may not have been strong enough to overcome the need to reduce cognitive dissonance (point three). We can speculate that there were more negative emotional responses than were reported and the reduction in cognitive dissonance works particularly well at the level of reporting. However, from the reported evidence, the emotional response to ICBO talks is overwhelmingly positive.

We described the emotional response to one talk in detail. Linking responses to times or events in the featured talk may break confidentiality, so the description below is only at the most general level. Participant 125 gave many responses (in time order): ‘joyful’, ‘this is in line with my ideals’, ‘contented’, ‘this is in line with my ideals’, ‘pleasure’, ‘good’, ‘this is important for my goals’, ‘this is pleasant’, ‘contented’, ‘this is expected’, ‘amused’, ‘contented’, ‘tired’, ‘proud’, ‘I am being treated justly’, ‘I am being treated justly’, ‘euphoric’, ‘this is in line with my ideals’ and ‘contented’. From this we may infer that he or she found the talk in line with their thinking and we could speculate that the participant was mentioned or his/her work was mentioned. Participants 23, 40 and 123 also expressed a similar emotional profile. For example, participant 40 was ‘interested’, ‘amused’, ‘energetic’, and thought ‘this is pleasant’.

‘Amused’, ‘I feel contented’, ‘good’ and ‘interested’ are among the most frequent emotional responses to this talk, which, assuming these 15 responses are indicative of the audience as a whole, means this talk was well received emotionally (though, as discussed, one may suspect that negative responses are less likely to be expressed). There were eight ‘amused’ responses from eight participants and seven of these were spread throughout the talk, suggesting an even level of amusement; one of the participants being amused two hours after the talk, suggesting either sustained amusement or a tardy response.

In contrast, Participant 6 had a different profile and was ‘bored’, ‘bored’, ‘tired’, ‘restless’ and ‘angry’ during the course of this talk. The response to this talk was generally positive emotionally, but the ability to discriminate between participants (as shown in the earlier analysis) is exhibited.

### Related work

#### Emotion self-reporting

Mood or emotion monitoring via questionnaires and self-reporting has been used in mental healthcare contexts, and more recently mobile phones have been adopted to serve that purpose [15]. Morris *et al.*[31] describe a mobile phone application developed to allow the self-monitoring of emotional state. The application prompted users to self-report their emotions several times a day, giving them a scale on which they could set either a single dimensional rating with different emotion types or a multidimensional “Mood Map” rating which allowed users to select a point on a valence vs. arousal graph. The single dimensional scales were offered for the emotion types happiness, sadness, anxiety, and anger. These emotion types are all present in the EM ontology. Compared to our tool, their tool offered a reduced number of distinct emotion types, with easier usability (using a touch interface). They coupled this experience sampling application with a mobile therapy utility that offered cognitive behavioural therapy via questions and suggested thought exercises designed to improve the well-being of the user through altering their reaction to common stressors. Reid *et al.*[32] developed a mobile phone application for self-reporting that offers a questionnaire to users on their mood, experiences and level of stress at times throughout the day. The application prompts users to collect data on mood and stress levels at four random times per day, and allows notes to be captured about locations and activities, correlated with usage of substances such as alcohol and cannabis. Mood capture used Likert scales in which adjectives indicating increasing degrees of the relevant mood were displayed on the phone screen rather than numbers. The focus was on negative moods, offering scales for angry, sad, tired, stressed, and anxious moods. Mood or emotion tagging smartphone applications that are commercially available and may be recommended for self-monitoring in cases of bipolar disorder include MoodTrak [33] and the T2 mood tracker [34]. MoodTrak allows free-text description of the present emotion being experienced coupled with a star rating (1–5) that ranks the mood from positive to negative. Tracking of moods is done online and a graphing facility shows a history. However, no private option is available, raising difficulties for confidential or sensitive usage scenarios. The use of free text to capture the name of the emotion being experienced hinders subsequent harmonization for research purposes of heterogeneous data arising from different users. The T2 mood tracker offers variable scales along which a rating can be selected. Pre-loaded scales include anxiety, stress, depression, brain injury, and general well-being. However, the scales are customizable.

It is our belief that an ontology such as the EM could benefit such applications as these by providing agreed-on standard categories for emotion self-reporting, localizing the vocabulary management function (which includes translation management) in one central community-agreed facility. However, many of the applications currently used for emotion capture allow only a very restricted vocabulary of emotion types, thus not capturing the broad range of different types of emotion that can be experienced and reported on, but also not requiring much by way of vocabulary management. Others use free text to enable the widest range of emotion types to be reported, but this approach sacrifices the facility for later data aggregation across different users and even different tools and may hinder subsequent interpretation and analysis.

#### Ontology evaluation

Tartir *et al.*, 2010 [12] distinguish several broad technical approaches to evaluation of ontologies, including logic-based approaches that use the knowledge encoded in axioms in the ontology to check for unsatisfiable classes, and feature-based approaches that rely on metrics about the content of the ontology, such as the percentage of classes that lack textual definitions. According to Brank *et al.*[1], ontology evaluation approaches can be divided into 1) those that compare the ontology to a “gold standard”, 2) those that compare the ontology to a source of data about the domain being modeled in the ontology, 3) those which involve human assessment according to a predefined set of criteria, and 4) those which involve the use of the ontology in a given application together with an evaluation of the results. Our approach follows the fourth strategy in that grouping, namely we have implemented an application that makes use of the ontology and built into the application the infrastructure to evaluate the ontology in use for that application. Similarly, [13] use a task-based approach to evaluate the Gene Ontology in use for the enrichment analysis of gene sets resulting from microarray experiments. As highlighted in [35],

Since users of ontologies will benefit from something that ontologies can ‘do’, research in ‘applied’ ontology has to be measured based on how well ontologies ‘do’ their tasks.

Our evaluation of the EM is notable as a rare example of a designed study on an ontology’s ‘fitness for purpose’. Most evaluations of biomedical ontologies tend to fall into the first and third groups above, if they are evaluated at all, and thus the work presented here is a contribution into the field of evaluation of biomedical ontologies.

### Limitations

In [1], several limitations of the ontology-evaluation-in-application-use paradigm are raised. Firstly, they point out that the evaluation of an ontology in a particular application can yield a result that only has scope for similar tasks. That is, in the context of the EM, our evaluation in use for self-reporting of emotional experiences in a conference can only inform the applicability of the ontology for self-reporting tasks (at academic conferences) and not for other types of use to which the ontology may be put. This is a fair point, and one that we are happy to concede as a limitation of the present study.

Secondly, they raise the concern that the ontology contribution to the overall application might be minimal compared to the remainder of the implementation, and that it can be difficult to separate the contribution of the ontology alone from the other aspects of the application. While our application design tried to minimise the effect of all but the most direct aspects of the application aside from the available vocabularies, we agree that there nevertheless might have been some impact on our results due to non-ontology-related aspects of the application. For example, the order in which words were presented in the selection boxes might have had some influence on the selected results and on the experienced ease of use. To control for these effects, however, we explicitly asked users to rate how easy it was for them to express their feelings using the vocabulary provided, rather than how easy the application as a whole was to use.

They further report that this paradigm cannot easily be used to compare different ontologies unless a single application can be reused with different pluggable ontologies. It was not our objective in the present study to compare different ontologies in the emotional domain, but we do believe that the application we have designed would be able to accommodate different sources of vocabulary should such an experiment be conducted in the future.

One clear limitation of our study is that only the vocabulary offered in the ontology has been evaluated, rather than the logical or hierarchical structure of the EM. Our findings thus only relate to the vocabulary component of the ontology, and a separate evaluation would be needed for the other aspects of the ontology. However, the study does show that the EM largely contained the vocabulary necessary for the participants to articulate their emotions.

Another limitation is that the study only evaluates the users’ *self-assessed* reports of their emotions, that is, the study makes no attempt to calibrate the reporting of emotions that the users provided with any objective psychometric evaluation of the emotions they were actually undergoing at that time. Our findings are thus only relevant to the self-reporting of self-assessed emotions, and not to the objective measuring of emotions as might be required in clinical settings. As the evaluation was intended to reveal whether the EM was sufficient to allow participants to self-report their emotional response, as opposed to revealing the true state of emotions at the ICBO 2012 conference, we do not see this as a significant issue in this evaluation.

A further limitation of the environment in which our study was conducted was that internet difficulties and power failures might have prevented some participants from recording their emotion effectively and the associated rating at the time that they would have liked. We did allow for post-hoc capture of tags to get around this problem, and we did see a spike of captures late at night (around 11pm) that was probably explained by this phenomenon. We did not optimise the appplication for use on mobile devices; this could have eased use and increased the number of users. Also, we were asking a lot of the conference attenders – many responses for many talks.

Finally, the emotional words from the EM that were used in the experiment themselves had implicit strengths which were not exposed in the analysis or correlated with the stated ‘strength of response’. This information was not available as an annotation in the EM ontology; however, it may be added in a future release.

## Conclusion

We find that the vocabulary provided by the leaves of the Emotion Ontology is suitable for use for the self-assessed self-reporting of emotional experiences in a conference setting. We evaluated the EM ‘in the wild’ in this setting and found that the EM can be used to discriminate between and articulate emotional experiences of audience members. We have released the EmOntoTag tool as open source for community adoption, adaptation and reuse in similar scenarios in future projects. We understand this experiment as a contribution to the ontology evaluation domain from a perspective of use-case driven evaluation, and we have been able to enhance the ontology based on the feedback and comments we received during the course of this experiment. Ontology evaluation is recognised to be a hard task that is not often performed and this work is a contribution of a formal experiment designed to evaluate the ability of an ontology’s vocabulary to make the distinctions necessary in a field of interest.

Future work will involve further adapting the ontology to allow more comprehensive descriptions of the context and causes for a particular emotional experience, and evaluating the ontology for use in the self-reporting of emotions in more clinical contexts, e.g. to facilitate emotional monitoring in the treatment of patients with mood disorders. There are a broad range of application scenarios in which the self-reporting of emotions might be relevant – almost any situation that has human involvement – and the EM is a candidate vocabulary for such applications.

## Electronic supplementary material

Additional file 1: **Sample user handout for ICBO conference participants.** Conference participants were given a handout together with their conference pack that detailed the experiment that we were conducting and assigned each user a different, unique, random access code for the system. An example of these handouts is included as a supplementary file. (PDF 69 KB)
