# Supplementary material for: Evaluating the Emotion Ontology through use in the self-reporting of emotional responses at an academic conference
Source: J Biomed Semantics. 2014 Sep 3;5:38. doi: 10.1186/2041-1480-5-38 (PMC4417517; doi:10.1186/2041-1480-5-38)
Supplement: Supplementary file 1 — Additional file 1: Sample user handout for ICBO conference participants. Conference participants were given a handout together with their conference pack that detailed the experiment that we were conducting and assigned each user a different, unique, random access code for the system. An example of these handouts is included as a supplementary file. (PDF 69 KB) [file 13326_2013_210_MOESM1_ESM.pdf]

Evaluating the Emotion Ontology at ICBO 2012 with EmOntoTag

Janna Hastings[1] and Robert Stevens[2]
[1]Swiss Center for Affective Sciences,
University of Geneva
Rue des Battoirs
1205 Geneva, Switzerland; and

[2]School of Computer Science
University of Manchester
Oxford Road
Manchester
United Kingdom
M13 9PL

We are running a study at ICBO 2012 to evaluate the Emotion Ontology[1]. We are doing this by finding out how well the ICBO 2012 audience can use the emotion ontology to articulate their emotional response to an ICBO 2012 presentation. The study uses a Web site called EmOntoTag and will work like this:

1. A user picks a talk in progress;
2. Users then complete sentences such as:

    'I feel *calm*';

    'I feel *happy*';

    'I think *there will be consequences*'.
3. For each sentence, a range of completion options for the *italicised parts* is available from the Emotion Ontology;
4. As many sentences can be made as required to articulate an emotional response to a presentation;
5. For each sentence, users can express the severity of their emotional response (with a 'weakly' to 'strongly' scale) and how easy it was to articulate their emotional response (via an 'easy' to 'difficult' scale);
6. The ease of articulation of overall emotional response is also captured for each presentation;
7. It is this 'ease of articulation' that is the primary aim of this study.

To express your emotional response to ICBO presentations go to:

`http://bioontology.ch/emontotag`

and enter the user identifier:

xxxxxxxx

Each user has a unique identifier. The EmOntoTag system has no record of any user's identity. The user identifiers above are automatically generated and not associated with any names. The user identifiers are only stored to discriminate people's responses from one another. All presentations available in this study have been given permission to be included by the presenters. As far as the talks are concerned, results are presented in an anonymous form. We are happy to give presenters feedback about their presentations, but we will not make any emotional responses public, including in publications, without the presenter's permission. The data are on a secure server and the data are encrypted.

For any details, queries or comments please email `hastings@ebi.ac.uk`.

[1]`http://code.google.com/p/emotion-ontology/`
